# First isolation and characterization of *Brucella microti* from wild boar

**DOI:** 10.1186/s12917-015-0456-z

**Published:** 2015-07-11

**Authors:** Zsuzsanna Rónai, Zsuzsa Kreizinger, Ádám Dán, Kevin Drees, Jeffrey T. Foster, Krisztián Bányai, Szilvia Marton, Levente Szeredi, Szilárd Jánosi, Miklós Gyuranecz

**Affiliations:** Veterinary Diagnostic Directorate, National Food Chain Safety Office, P.O. Box 2, 1581 Budapest, Hungary; Institute for Veterinary Medical Research, Centre for Agricultural Research, Hungarian Academy of Sciences, Hungária körút 21, 1143 Budapest, Hungary; University of New Hampshire, Durham, New Hampshire USA

**Keywords:** Biochemistry, *Brucella microti*, Immunohistochemistry, MLVA, Morphology, Wild boar, Whole genome sequencing, Hungary

## Abstract

**Background:**

*Brucella microti* was first isolated from common vole (*Microtus arvalis*) in the Czech Republic in Central Europe in 2007. As *B. microti* is the only *Brucella* species known to live in soil, its distribution, ecology, zoonotic potential, and genomic organization is of particular interest. The present paper is the first to report the isolation of *B. microti* from a wild boar (*Sus scrofa*), which is also the first isolation of this bacterial species in Hungary.

**Results:**

The *B. microti* isolate was cultured, after enrichment in *Brucella*-selective broth, from the submandibular lymph node of a female wild boar that was taken by hunters in Hungary near the Austrian border in September 2014. Histological and immunohistological examinations of the lymph node sections with *B. abortus-*, *B. suis-* and *B. canis*-specific sera gave negative results. The isolate did not require CO_2_ for growth, was oxidase, catalase, and urease positive, H_2_S negative, grew well in the presence of 20 μg/ml basic fuchsin and thionin, and had brownish pigmentation after three days of incubation. It gave strong positive agglutination with anti-A and anti-M but had a negative reaction with anti-R monospecific sera. The API 20 NE test identified it as *Ochrobactrum anthropi* with 99.9 % identity, and it showed *B. microti*-specific banding pattern in the Bruce- and Suis-ladder multiplex PCR systems. Whole genome re-sequencing identified 30 SNPs in orthologous loci when compared to the *B. microti* reference genome available in GenBank, and the MLVA analysis yielded a unique profile.

**Conclusions:**

Given that the female wild boar did not develop any clinical disease, we hypothesize that this host species only harboured the bacterium, serving as a possible reservoir capable of maintaining and spreading this pathogen. The infectious source could have been either a rodent, a carcass that had been eaten or infection occurred via the boar rooting in soil. The low number of discovered SNPs suggests an unexpectedly high level of genetic homogeneity in this *Brucella* species.

## Background

The genus *Brucella* gained numerous new members in recent years. *Brucella ceti* and *B. pinnipedialis* have been isolated since the mid-1990’s from marine mammals but were only named and designated in 2007 [1]. *B. inopinata* was described in 2010 [2], ‘atypical’ *Brucella* strains from frogs in 2012 and 2015 [3, 4], and the most recent member of the genus is *B. papionis*, which was isolated from baboons [5].

Hubalek et al. [6] reported brucellosis in common voles (*Microtus arvalis*) in the Czech Republic, and the pathogen was described as *B. microti* [7]. Since then it has been detected in soil samples [8] and lymph nodes of red foxes [9] in the Czech Republic and Austria. The whole genome sequence of the type strain CCM 4915^T^ was published in 2009 [10], its pathogenic potential was investigated in murine models [11], and the intraspecies biodiversity of the species was investigated by Al Dahouk and colleagues [12]. As *B. microti* is the only *Brucella* species known to persist in soil, it is of particular interest as an environmental reservoir and data on the distribution, ecology, zoonotic potential, genomic organization of this bacterium and relatedness to other strains are urgently needed [12].

A *B. microti* strain was isolated from the submandibular lymph node of a female wild boar (*Sus scrofa*) in Hungary near Rajka town (47.9 °N, 17.2 °E) in close vicinity to the Austrian border in September, 2014. This is the first isolation of *B. microti* from wild boar. The aim of the study was to describe the isolation conditions and to determine the morphological, biochemical, and genetic characteristics of the isolate and compare it with previous *B. microti* isolates originating from different host species.

## Methods

The submandibular lymph node of a hunted female wild boar was collected as part of the national wildlife health monitoring programme and submitted for routine diagnostic examination, therefore ethical approval was not required for the study. After gross pathological examination, half of the lymph node was fixed in 8 % formalin and embedded in paraffin for hematoxylin-eosin, and immunohistochemical examinations as described previously [13]. In brief, 4-μm thick deparaffinized serial sections were incubated overnight at 4 °C with *B. abortus-*specific (S type *Brucella* species’), *B. canis*-specific (R type *Brucella* species’) hyperimmune rabbit serum or *B. suis*-specific (S type *Brucella* species’) hyperimmune mouse serum diluted to 1:100.000, 1:20.000, and 1:40.000 respectively, and antibody binding was detected by a horseradish peroxidase–labeled polymer (EnVision + Kit, Dako Denmark A/S, Glostrup, Denmark).

The other half of the lymph node was sliced up and mixed with 10 ml phosphate buffered saline, then homogenized with a laboratory blender (5 min at blending speed of 10 strokes/sec; BagMixer 400 VW, InterScience, Saint Nom, France). Selective *Brucella* agar plates (Oxoid Ltd., Cambridge, United Kingdom) containing 5 % heat inactivated horse serum (Invitrogen Corp., Carlsbad, CA) and *Brucella* selective supplement (Oxoid Ltd., Cambridge, United Kingdom) were inoculated with 100 μl of homogenized tissue. The plates were incubated at 37 °C in 5 % CO_2_ for 10 days. In addition, 1 ml aliquot of homogenized lymph node was cultured at 37 °C for 10 days in *Brucella* selective broth with *Brucella* selective supplement (Oxoid Ltd., Cambridge, United Kingdom) before 100 μl of inoculum was transferred to *Brucella* agar plates as described above and incubated under the same conditions. Growth of *Brucella* was checked daily. Routine biochemical and growth-based typing tests like parallel incubation with and without CO_2_ were performed on the isolates [14]. API 20 NE test (BioMérieux, Marcy l’Etoile, France) was performed according to the manufacturer’s instructions. Slide agglutination tests of the isolated strain was carried out using A, M (monospecific sera against the A or M agglutinogen of *Brucella* spp. forming smooth /S/ colonies), and R (serum against *Brucella* spp. with rough /R/ colony form) sera (French Agency for Food, Environmental & Occupational Health Safety /ANSES/, Maisons-Alfort Cedex, France).

DNA was extracted with the QIAamp DNA Mini Kit (Qiagen Inc., Valencia, CA) from 1 distinct colony and the upgraded Bruce-ladder and Suis-ladder PCR systems were used for the molecular identification of the isolated strain [15]. Next-generation sequencing technology was used to re-sequence the genome of the isolate. One hundred ng of DNA was subjected to enzymatic fragmentation using the reagents supplied in the NEBNext® Fast DNA Fragmentation & Library Prep Set for Ion Torrent™ kit (New England Biolabs, Hitchin, United Kingdom) according to the manufacturer’s instructions. The adaptor ligation was performed using reagents from the same kit, whereas barcoded adaptors were retrieved from the Ion Xpress™ Barcode Adapters (Life Technologies Inc., Waltham, MA, USA). The barcoded library DNA samples were column purified using the Gel/PCR DNA Fragments Extraction kit (Geneaid Biotech Ltd., Taipei, Taiwan). Then the eluted library DNA was run on 2 % precast gel. Products between 300 and 350 bp were directly used without further purification in the PCR mixture of the NEBNext® Fast DNA Fragmentation & Library Prep Set for Ion Torrent™ kit (New England Biolabs). Library amplification involved 12 amplification and the products were purified by the Gel/PCR DNA Fragments Extraction kit (Geneaid). The DNA was eluted in 50 μl nuclease free water and quantified fluorometrically on Qubit® 2.0 equipment using the Qubit® dsDNA BR Assay kit (Invitrogen). The appropriately diluted library DNA was used in emulsion PCR. This step was carried out according to the manufacturer’s protocol using the Ion PGM Template kit on OneTouch v2 instrument. Enrichment of the templated beads (on Ion One Touch ES machine) and further steps of pre-sequencing set-up were performed according to the 200 bp protocol of the manufacturer. The sequencing protocol recommended for Ion PGM™ Sequencing Kit on a 316 chip was strictly followed.

Sequence data were curated and mapped onto the reference *B. microti* chromosome sequences (NC_013119, NC_013118) obtained from GenBank using the Lasergene Genomics Suite Software (DNASTAR Inc., Madison, WI, USA) and putative single nucleotide polymorphisms (SNPs) were identified between the two genomes under the following settings; mer size: 19 nt, minimum match percentage: 93, minimum alignment length: 25, maximum gap size: 30, match score:10, mismatch penalty: 20, gap penalty: 50, gap extension penalty: 10, alignment cutoff: 200, SNP calculation method: haploid bayesian, P not reference percentage: 75 %, Phred score (Q call): 30, SNP percentage: 75–100 %, coverage depth minimum: 5. Validity of SNPs were confirmed by visual examination in the assembled sequences.

Multiple-locus variable-number tandem repeat analysis (MLVA) based on 16 variable-number tandem repeats (VNTR) (MLVA16) was performed on the isolated strain as described previously using singleplex PCR amplifications [16–18]. The reference strain *B. suis* Thomsen (ATCC 23445^T^) was used as a standard during the amplifications to confirm appropriate fragment size estimations. The number of repeats was estimated from the amplicon sizes according to the 2013 *Brucella* allele assignment table (http://mlva.u-psud.fr). As the application of the MLVA-11 (including only loci from panels 1 and 2A) subset was found to be more useful in the comparison of strains with different origin than MLVA16 [19, 17], the evolutionary relatedness among the allelic profiles of the present isolate and *B. microti* strains available in the *Brucella* MLVA database was examined with the goeBURST algorithm for the compilation of minimum spanning tree in Phyloviz based on MLVA11 data [20]. The genotype of the reference strain of *B. suis* biovar. 5 (NCTC11996^T^) (http://mlva.u-psud.fr) was also included in the analysis as an outgroup.

## Results

The submandibular lymph node sample of the wild boar did not show any gross pathological or histological changes and was negative with *B. abortus*, *B. suis* and *B. canis* specific sera in the immunohistochemical examinations. The directly inoculated *Brucella* selective agar remained negative during the incubation period but colonies appeared in pure culture after two days incubation on the *Brucella* selective agar inoculated with the enriched *Brucella* selective broth. The colonies were small, translucent, had creamy consistency and displayed a brownish pigmentation after 3 days of incubation. The Gram-negative and modified acid-fast, small coccobacilli demonstrated oxidase, catalase, and urease activity, but failed to produce H_2_S. The isolate grew in the presence of 20 μg/ml thionin and basic fuchsin and did not require CO_2_ for its growth. The isolate showed strong agglutination with sera A and M, but agglutination was not observed with serum R. The API 20 NE test identified it as *Ochrobactrum anthropi* with 99.9 % identity, with positive reactions in nitrate reduction, urease activity, D-glucose, L-arabinose, D-mannose, N-acetyl-glucosamine, D-maltose, adipic acid and malic acid assimilation tests and negative reactions for indole production, D-glucose fermentation, arginine dihidrolase tests, esculin and gelatin hydrolysis, β-galactosidase activity, and in D-mannitol, potassium gluconate, capric acid, trisodium citrate and phenylacetic acid assimilation reactions.

**Fig. 1:**
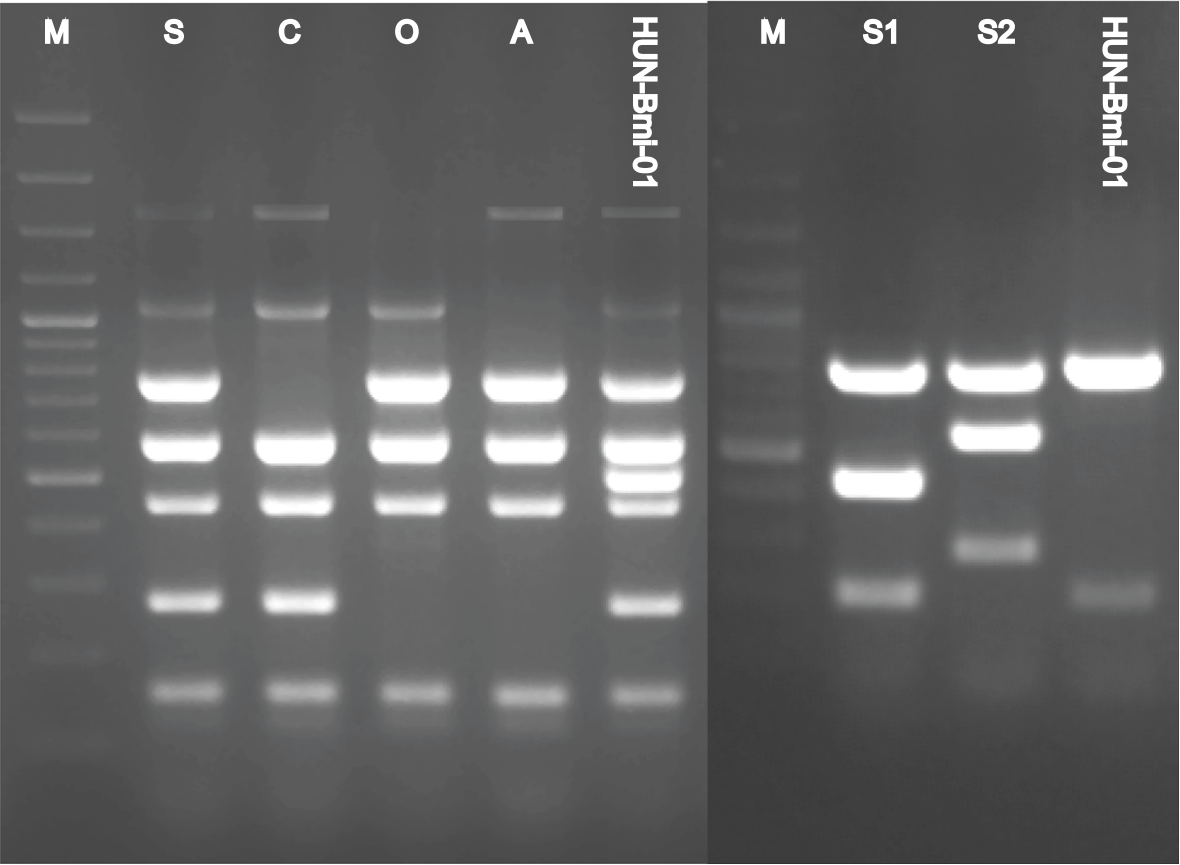
Results of upgraded “Bruce- and Suis-ladder” PCRs of HUN-Bmi-01 strain. **left side** Bruce-ladder v2.0 PCR with 1682, 1071, 774, 587, 510, 450, 272, and 152 bp amplicons. M: 100 bp DNA marker, S: *B. suis*, C: *B. canis*, O: *B. ovis*, A: *B. abortus*. **right side** Suis-ladder PCR with 774 and 197 bp amplicons. M: 100 bp DNA marker, S1: *B. suis* biovar. 1, S2: *B. suis* biovar. 2

**Fig. 2:**
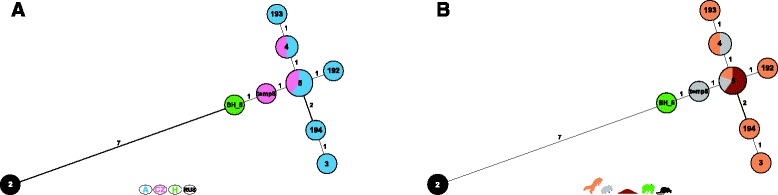
Minimum spanning tree analysis of *Brucella microti* isolates using multiple-locus variable-number tandem repeat analysis (MLVA-11 subset) data. The numbers and identification codes (BH_5: HUN-Bmi-01; temp8: *in silico* data from CCM 4915^T^) represent the 9 MLVA-11 genotypes examined in this study. The size of the shapes indicates the number of isolates sharing the genotype. Branch lengths and numbers are corresponding to the level of genetic distances between the genotypes. Colour codes are associated with the country of origin (A) and the host species of origin (B) of *B. microti* genotypes. Codes of country of origin (A): Austria (A, *blue*), Czech Republic (CZ, *pink*), Hungary (H, *green*), and former USSR (RUS, *black*). Colours indicate the host species of origin as follows (B): red fox (*orange*), common vole (*grey*), soil (*brown*), wild boar (*green*), and wild rodent (*black*; reference strain *B. suis* biovar. 5)

The isolate was identified as *B. microti* (strain ID: HUN-Bmi-01) with the upgraded “Bruce- and Suis-ladder” methods. In Bruce-ladder v2.0 1682, 1071, 774, 587, 450, 272, 152, and the *B. microti* specific 510 bp fragments were amplified, while in Suis-ladder 774 and 197 bp amplicons appeared (Fig. 1). The whole genome sequence of the isolate was deposited in GenBank [Accession number SRP053188]. The number of reads used for mapping was 2,705,699 (1,725,428 on chromosome 1 and 980,271 on chromosome 2), while 122,318 reads were discarded during alignment. The median coverage was 142X on chromosome 1 and 140X on chromosome 2. In the whole genome sequence, 19 SNPs were identified on chromosome 1 and 11 SNPs on chromosome 2 compared to the *B. microti* reference genome available in GenBank (Table 1). For the 30 SNPs, 6 were in intergenic regions (non-coding) and 24 were within known gene regions; 15 of intragenic SNPs were synonymous mutations and 9 were non-synonymous. The MLVA16 analysis yielded a novel profile (BH_5: 4-5-12-13-5-2-5-6-7-7-9-10-13-12-4-6, in order of loci Bruce06-08-11-12-42-43-45-55-18-19-21-04-07-09-16-30) compared to the other 11 *B. microti* strains available in the MLVA bank. The genetic relatedness of the strains based on the MLVA11 subset is displayed on Fig. 2.

**Table 1:** SNPs identified in the new *B. microti* isolate

Chromosome	Reference sequence ID	Position in reference sequence	Reference base	Base in the studied *B. microti* strain (HUN-Bmi-01)	Location of mutation	Impact of mutation	SNP %	P not reference value	Phred score	Coverage depth
I	NC_013119	63814	A	G	BMI_I59	synonymous	99.1 %	100.0 %	60.000	119
I	NC_013119	115266	C	A	cysA	non-synonymous	96.4 %	100.0 %	60.000	84
I	NC_013119	220835	G	T	BMI_I210	non-synonymous	100.0 %	100.0 %	60.000	140
I	NC_013119	300834	G	A	fadD	synonymous	99.3 %	100.0 %	60.000	160
I	NC_013119	362076	T	C	xdhB	synonymous	99.2 %	100.0 %	60.000	126
I	NC_013119	530242	N	A	BMI_I534	synonymous	86.1 %	100.0 %	60.000	94
I	NC_013119	530273	N	A	BMI_I534	non-synonymous	75.4 %	100.0 %	39.876	106
I	NC_013119	549069	G	A	intergenic region		100.0 %	100.0 %	60.000	109
I	NC_013119	643213	T	C	intergenic region		99.2 %	100.0 %	60.000	134
I	NC_013119	806268	A	G	intergenic region		98.0 %	100.0 %	60.000	153
I	NC_013119	1078051	T	C	gyrA	non-synonymous	100.0 %	100.0 %	60.000	146
I	NC_013119	1141643	C	T	BMI_I1167	synonymous	100.0 %	100.0 %	60.000	123
I	NC_013119	1145284	C	T	pyrH	non-synonymous	82.1 %	100.0 %	60.000	95
I	NC_013119	1251949	G	T	BMI_I1296	synonymous	100.0 %	100.0 %	60.000	23
I	NC_013119	1252016	A	T	BMI_I1296	non-synonymous	100.0 %	100.0 %	60.000	13
I	NC_013119	1778954	G	A	BMI_I1855	synonymous	95.2 %	100.0 %	60.000	146
I	NC_013119	1790391	T	G	BMI_I1868	synonymous	93.1 %	100.0 %	60.000	132
I	NC_013119	1790394	C	A	BMI_I1868	synonymous	94.0 %	100.0 %	60.000	152
I	NC_013119	1853511	C	T	BMI_I1931	non-synonymous	98.8 %	100.0 %	60.000	171
II	NC_013118	109915	A	G	BMI_II112	synonymous	99.4 %	100.0 %	60.000	178
II	NC_013118	483021	G	A	BMI_II495	synonymous	100.0 %	100.0 %	60.000	174
II	NC_013118	689243	A	G	intergenic region		100.0 %	100.0 %	60.000	11
II	NC_013118	739665	C	A	intergenic region		100.0 %	100.0 %	60.000	148
II	NC_013118	816002	C	T	BMI_II819	synonymous	92.2 %	100.0 %	60.000	90
II	NC_013118	834372	T	C	BMI_II841	synonymous	97.8 %	100.0 %	60.000	232
II	NC_013118	834392	N	G	BMI_II841	synonymous	98.0 %	100.0 %	60.000	204
II	NC_013118	951779	G	C	intergenic region		80.1 %	100.0 %	60.000	563
II	NC_013118	978988	C	A	BMI_II983	synonymous	98.3 %	100.0 %	60.000	124
II	NC_013118	1186061	C	T	BMI_II1178	non-synonymous	100.0 %	100.0 %	60.000	138
II	NC_013118	1186062	C	T	BMI_II1178	non-synonymous	100.0 %	100.0 %	60.000	138

This table displays the SNPs witch were identified during the whole-genome comparison of the studied *B. microti* isolate (HUN-Bmi-01, [GenBank: SRP053188]) with the reference genome (strain CCM 4915 ^T^, NC_013119, NC_013118), with information on their location, impact and quality

## Discussion

The hunting area in Hungary where the wild boar was harvested is close to Austria where several previous *B. microti* isolates originated. Hungary is located in Central Europe and current data indicate that countries in this area are enzootic foci of *B. microti*. We hypothesize that the wild boar acquired the *B. microti* strain while eating a rodent, a carcass (e.g., dead fox), or simply rooting in the soil. The hunters did not report any gross pathological lesions in other parts of the carcass, so it seems likely that the wild boar did not develop any obvious clinical disease. Thus we suspect that wild boar merely serves as a reservoir species capable of maintaining and spreading the pathogen in nature.

The negative results of both the S and R type *Brucella*-specific immunohistochemical reactions imply that the amount of *B. microti* in the lymph node was below the detection limit of this method, which is also supported by the finding that the strain could only be isolated after enrichment in *Brucella* selective broth. These results and the lack of histological lesions in lymph node further support the hypothesis that the wild boar was only an asymptomatic carrier of this bacterium.

The morphological, growth and biochemical characteristics of the isolated *B. microti* strain were congruent with the description of Scholz et al. [7], including the described brownish pigmentation of the colonies after 3 days incubation. The isolate displayed a unique agglutination reaction, agglutinating with anti-A and anti-M but not with anti-R monospecific sera. The first common vole strains only agglutinated with anti-M serum [7], but Al Dahouk et al. [12] reported several different agglutination patterns; despite the M antigen dominancy a fox isolate only agglutinated with anti-A serum, while the soil isolates agglutinated all three monospecific sera.

The observed 30 SNPs between the whole genome sequence of the Czech common vole and Hungarian wild boar strain is surprisingly few, suggesting relatively high genetic homogeneity within *B. microti* in Central Europe. We omitted potential sources of variation such as multi-copy genetic elements, insertion sequences, and repeat regions from the SNP analysis. Thus, our estimate of the number of SNPs is a conservative one but is more likely to represent biologically meaningful differences. Moreover, SNPs from homologous loci have been demonstrated as reliable for phylogenetic analysis of *Brucella* species because of their coverage of the entire genome, relative stability over evolutionary time, ease of comparison, and inclusion of intergenic regions [21]. Therefore the primary aim of SNP analysis was to phylogenetically (at large evolutionary scale level) compare the present Hungarian *B. microti* isolate with the Czech *B. microti* strain.

The highly mutable genetic markers, VNTRs, are not captured in the re-sequenced draft genome. However, our amplicon sequencing of these loci allowed for placement of our isolate within a MLVA framework, indicating that the Hungarian wild boar *B. microti* isolate is clearly differentiated from the common vole, fox and soil isolates originated from Austria and the Czech Republic. The question regarding the source of infection in wild boar (i.e., common vole, fox, soil or other host) remains open and additional efforts to better understand the epidemiology and ecology of *B. microti* are needed.

## Conclusion

This is the first isolation of *Brucella microti* from a wild boar, and also the first isolation of this species in Hungary. The information gained through bacteriological, histological, immunohistological and molecular analysis with emphasis on whole genome re-sequencing highly contribute to our knowledge of the host range, geographic distribution and genomic organization of *B. microti*.

## Abbreviations

MLVA: Multiple-locus variable number tandem repeat analysis
SNP: Single nucleotide polymorphism
VNTR: Variable number tandem repeats
